# Brain network representations of placebo analgesia

**DOI:** 10.1017/S0033291726103924

**Published:** 2026-05-05

**Authors:** Xiaohan Zhang, Xuetian Sun, Weisheng Huang, Kaijie An, Xufeng Zhao, Dan Zhang, Wenwei Zhang, Yongqiang Yu, Yinfeng Qian, Jiajia Zhu

**Affiliations:** 1 Department of Radiology, The First Affiliated Hospital of Anhui Medical University, Hefei, China; 2 Research Center of Clinical Medical Imaging, Anhui Province, Hefei, China; 3 Anhui Provincial Key Laboratory for Brain Bank Construction and Resource Utilization, Hefei, China

**Keywords:** brain network, functional connectivity, neuroimaging, pain, placebo analgesia

## Abstract

**Background:**

Prior neuroimaging studies and meta-analyses investigating brain correlates of placebo analgesia (PA) have yielded neuroanatomically heterogeneous findings, which may be reconciled from a connectomics perspective. The objective of this study was to examine network localization of brain functional alterations related to PA.

**Methods:**

We initially identified PA-induced brain activation alterations (hyper-activation and hypo-activation separately) during experimental pain from 29 published studies with 674 individuals. By combining these implicated dysfunctional brain regions with large-scale discovery (N = 1113) and validation (N = 1093) resting-state functional magnetic resonance imaging datasets, we then employed a novel functional connectivity network mapping approach to construct PA hyper-activation and hypo-activation networks, respectively.

**Results:**

The PA hyper-activation network manifested as a pattern of circumscribed brain regions mainly involving the limbic, default, and frontoparietal networks. By contrast, the PA hypo-activation network comprised a broadly distributed set of brain regions primarily implicating the ventral attention, somatomotor, and subcortical networks.

**Conclusions:**

Our findings regarding the brain network representations of PA may contribute to a deeper understanding of its action mechanisms and provide a neural framework that may inform future clinical translation.

## Introduction

Placebo effects contribute substantially to treatment outcomes in both medical research and clinical practice. Placebo analgesia (PA), one of the most robust and best-studied placebo effects (Enck, Bingel, Schedlowski, & Rief, 2013; Hróbjartsson & Gøtzsche, 2010; Vase, Petersen, Riley, & Price, 2009), refers to the phenomenon that a belief or an expectation that one is receiving an effective analgesic treatment can actually reduce pain (Benedetti, Arduino, & Amanzio, 1999; Price, 2000). PA has been linked with multiple psychological processes, including expectations and beliefs (Atlas & Wager, 2012), associative learning (Zunhammer et al., 2017), and social cognition (Colloca & Benedetti, 2009). From a contemporary theoretical perspective, these psychological processes are increasingly understood through the framework of the Bayesian brain hypothesis (Büchel, Geuter, Sprenger, & Eippert, 2014; Kaptchuk, Hemond, & Miller, 2020), which is instrumental in evaluating the mechanisms of placebo effects (Pagnini et al., 2023; Poublan-Couzardot & Talmi, 2024). This framework posits that perception arises from a Bayesian integration process: top-down prior beliefs (such as treatment expectations) are combined with bottom-up sensory evidence, with both weighted by their relative precision to form the perceptual inference (de Lange, Heilbron, & Kok, 2018; Friston, 2010). This predictive coding account has been successfully applied to explain pain modulation by treatment expectations, including placebo hypoalgesia (Crawford et al., 2025; Grahl, Onat, & Büchel, 2018). Neurophysiological studies have suggested the involvement of descending inhibition of nociceptive afferents, with some studies supporting influences on spinal mechanisms and others supporting higher-level cortical effects that cannot be explained by nociceptive input modulation alone (Eippert et al., 2009; Geuter & Büchel, 2013; Goffaux, Redmond, Rainville, & Marchand, 2007; Matre, Casey, & Knardahl, 2006; Tinnermann et al., 2017), indicating affective or evaluative mechanisms (Martini, Lee, Valentini, & Iannetti, 2015; Petrovic et al., 2010; Wager, Atlas, Leotti, & Rilling, 2011; Wager, Matre, & Casey, 2006). Notwithstanding this, there is still an urgent need for a more sophisticated understanding of the neural mechanisms underlying PA, in order to optimize pain treatment strategies.

Functional neuroimaging techniques, such as functional magnetic resonance imaging (fMRI) and positron emission tomography (PET), have provided a powerful framework that allows for the study of PA-induced brain functional alterations. Leveraging these techniques, numerous studies have demonstrated that some brain regions including the prefrontal cortex, anterior cingulate cortex, insula, thalamus, striatum, and brainstem are frequently involved in the neural processes of PA (Bingel et al., 2006; de la Fuente-Fernández et al., 2001, 2002; Dum, Levinthal, & Strick, 2016; Grinband, Hirsch, & Ferrera, 2006; Jarcho et al., 2016; Leknes et al., 2011; Lindquist et al., 2012; Martikainen et al., 2005; Pariente, White, Frackowiak, & Lewith, 2005; Peciña et al., 2014; Petrovic et al., 2005; Petrovic, Kalso, Petersson, & Ingvar, 2002; Scott et al., 2007). Nevertheless, previous investigations have yielded inconsistent findings, and the lack of large sample assessments hampers the detection of small to moderate effects (Cremers, Wager, & Yarkoni, 2017). While several coordinate-based neuroimaging meta-analyses have focused on identifying the convergent neural representations of PA using a larger sample of participants pooled across studies (Amanzio et al., 2013; Atlas & Wager, 2014; Fu et al., 2021; Zunhammer, Spisák, Wager, & Bingel, 2021), the results also vary considerably and suggest the widespread nature along with two opposite patterns (hyper-activation and hypo-activation) of PA-induced regional brain functional changes, pointing toward a potentially more refined network-level mechanism.

The human brain is an intricate network of functionally specialized regions interconnected by white matter tracts, referred to as a connectome. As brain connectomics has evolved into a major concept in modern neuroscience, there is increasing convergent evidence that neuroanatomically heterogeneous findings in brain disorders can be reconciled from a connectomics perspective, that is, they localize to highly robust and clinically meaningful distributed brain networks rather than to individual brain regions (Fornito, Zalesky, & Breakspear, 2015; Fox, 2018). The network-based conceptualization of brain disorders has recently led to the revolutionary notion that therapeutic interventions for brain disorders also show network-level brain representations and yield clinical benefits in a network-dependent manner (Scangos et al., 2023; Siddiqi et al., 2021; Sobesky et al., 2022). To achieve network localization, a novel functional connectivity network mapping (FCNM) approach has been developed to map regional brain changes associated with a disease or a treatment to a common network based on the human brain functional connectome (Cheng et al., 2025; Mo et al., 2024; Xu et al., 2024; Zhang et al., 2024). Taking advantage of the FCNM approach, much effort has gone toward identifying specific brain networks in relation to certain brain disorders and therapeutic interventions (Darby, Joutsa, & Fox, 2019; Fox et al., 2014; Jones et al., 2023; Padmanabhan et al., 2019; Siddiqi et al., 2020; Taylor et al., 2023; Trapp et al., 2023; Zhukovsky et al., 2021), facilitating a better understanding of the disease and treatment mechanisms. Despite this progress, relatively little is known about network localization of brain functional alterations induced by PA.

**Figure 1.**
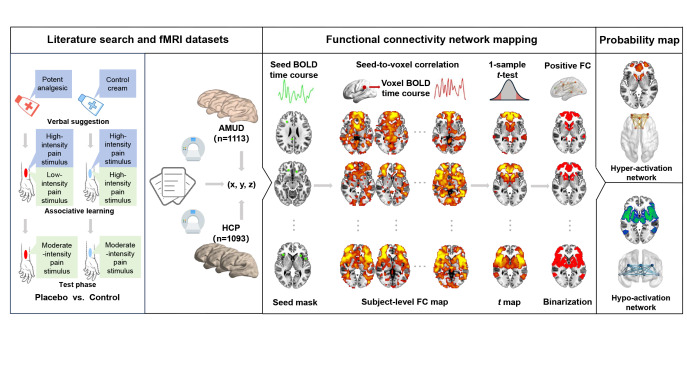
Overview of the study design and analyses. We initially synthesized published functional neuroimaging literature relevant to PA, with an emphasis on findings of PA-induced brain activation alterations (hyper-activation [placebo > control contrast] and hypo-activation [placebo < control contrast] separately) during experimental pain stimulus paradigms. By combining these implicated dysfunctional brain regions with large-scale resting-state fMRI datasets (the AMUD and HCP), we then employed the FCNM approach to construct PA hyper-activation and hypo-activation networks, respectively. Specifically, spheres centered at each coordinate of a contrast were firstly created and merged together to generate a contrast-specific combined seed mask. Second, based on the resting-state fMRI data, we computed a contrast seed-to-whole brain FC map for each subject. Third, the subject-level FC maps were entered into a voxel-wise one-sample *t*-test to identify brain regions functionally connected to each contrast seed. Fourth, the resulting group-level *t* maps were thresholded and binarized. Finally, the binarized maps were overlaid to produce two network probability maps, which were thresholded at 60% to yield the PA hyper-activation and hypo-activation networks, respectively. *Note*: AMUD, Anhui Medical University Dataset; BOLD, blood oxygen level dependent; FC, functional connectivity; FCNM, functional connectivity network mapping; fMRI, functional magnetic resonance imaging; HCP, Human Connectome Project; PA, placebo analgesia.

To address this missing gap, we initially synthesized published functional neuroimaging literature relevant to PA, with an emphasis on findings of PA-induced brain activation alterations (hyper-activation and hypo-activation separately) during experimental pain stimulus paradigms. By combining these implicated dysfunctional brain regions with large-scale discovery and validation resting-state fMRI datasets, we then employed the FCNM approach to construct PA hyper-activation and hypo-activation networks, respectively. An overview of the study design and analyses is illustrated in Figure 1.

## Materials and methods

### Study search and selection

We identified relevant studies investigating PA-induced alterations (within-subject placebo vs. control) in brain activation during experimental pain stimuli. A literature search was performed from January to March of 2024 in ScienceDirect and PubMed according to the Preferred Reporting Items for Systematic Reviews and Meta-Analyses (PRISMA) guidelines. The following key terms were used: [‘Placebo effect’ OR ‘Placebo analgesia’] AND [‘fMRI’ OR ‘functional magnetic resonance imaging’ OR ‘PET’ OR ‘positron emission tomography’]. For completeness, we searched the published meta-analyses and reviews, screened several other sources (e.g., references of the pre-selected articles), and used Google Scholar to identify additional relevant studies.

**Figure 2.**
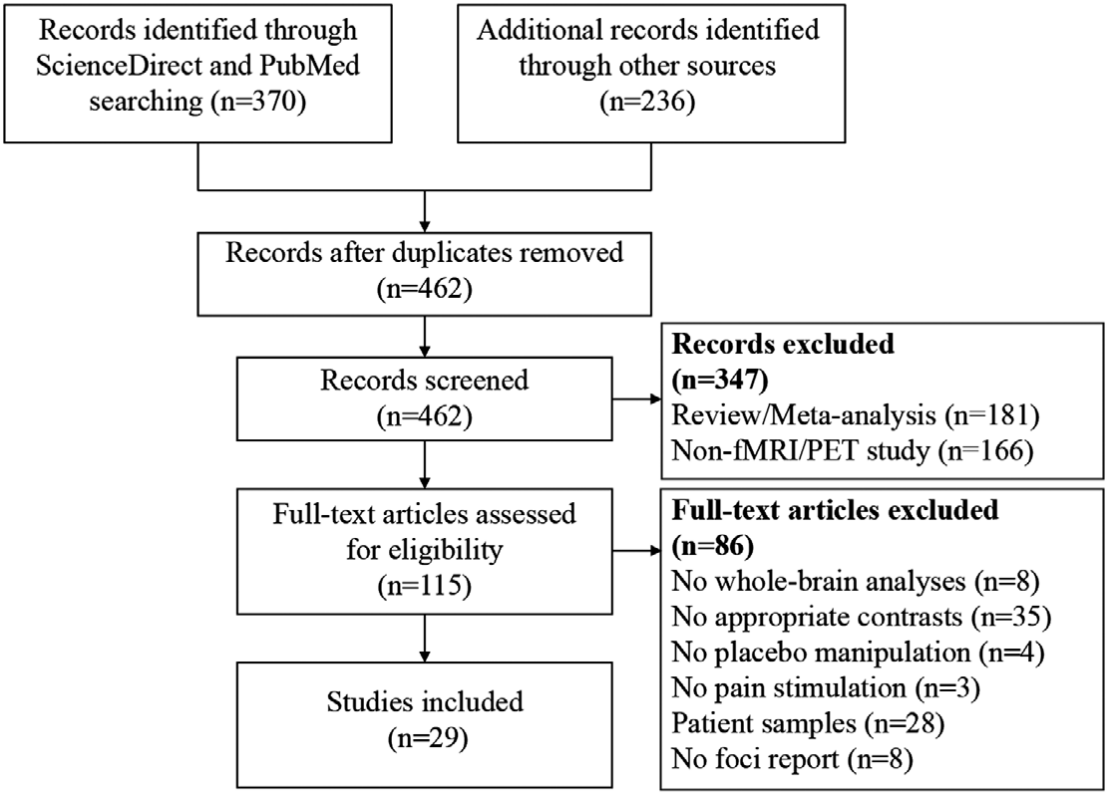
A flow diagram of the study selection process. *Note*: fMRI, functional magnetic resonance imaging; PET, positron emission tomography.

Eligible studies fulfilled the following inclusion criteria: 1) reporting the placebo versus control contrast using the whole-brain data with random effects analyses; 2) reporting coordinates of significant brain clusters in a standard reference space (Talairach or Montreal Neurological Institute [MNI] space); 3) using pain stimulation to induce pain; 4) reporting placebo manipulation; 5) carrying out research in healthy human participants. Studies were excluded based on the following criteria: 1) studies did not perform voxel-based analyses; 2) studies did not report results in Talairach or MNI space; and 3) studies were reviews or meta-analyses. Study search and selection were independently performed by two investigators. A flow diagram of the study selection process is shown in Figure 2. This protocol was registered on PROSPERO (https://www.crd.york.ac.uk/PROSPERO/, registration number: CRD42024557896). Peak coordinates of significant brain clusters with PA-induced hyper-activation (placebo > control contrast) and hypo-activation (placebo < control contrast) were extracted and analyzed, separately. Coordinates in Talairach space were converted to MNI space.

### Discovery and validation datasets

Our study used the Anhui Medical University Dataset (AMUD) as a discovery dataset and the Human Connectcome Project (HCP) as a validation dataset. The AMUD included 1113 healthy adults of Han Chinese (643 female, mean age = 32.66 ± 12.78 years), who were enrolled from local universities and communities through poster advertisements. Participants with neurological, psychiatric, or severe somatic disorders, a history of head injury with consciousness loss, MRI contraindications, or a family history of psychiatric diseases among their first-degree relatives were excluded. This study was approved by the ethics committee of The First Affiliated Hospital of Anhui Medical University, and all participants provided written informed consent after being given a complete description of the study. The HCP dataset is available for download to anyone agreeing to the open access data use terms (https://db.humanconnectome.org/). A total of 1093 healthy young adults (594 female, mean age = 28.78 ± 3.69 years) from the HCP were included in the validation dataset. The full details regarding the sample have been reported in prior publications (Glasser et al., 2016; Van Essen et al., 2012, 2013). The HCP protocol was approved by the Institutional Review Board of Washington University in St. Louis, MO, USA, and written informed consent was obtained from each participant. Demographic information of the discovery and validation datasets is provided in Table S1 in the Supplementary Materials.

### fMRI data acquisition and preprocessing

Resting-state fMRI data of the AMUD were collected on a 3.0-Tesla General Electric Discovery MR750w scanner and those of the HCP dataset on a 3.0-Tesla Siemens Skyra scanner. The fMRI parameters of the two datasets are provided in Table S2 in the Supplementary Materials. Participants with poor image quality (e.g., visible artifacts) or incomplete brain coverage were excluded.

The AMUD resting-state fMRI data were preprocessed using Statistical Parametric Mapping software (SPM12, http://www.fil.ion.ucl.ac.uk/spm) and Data Processing & Analysis for Brain Imaging (DPABI, http://rfmri.org/dpabi) (Yan, Wang, Zuo, & Zang, 2016). The first 10 volumes for each participant were discarded to allow the signal to reach equilibrium and the participants to adapt to the scanning noise. The remaining volumes were corrected for the acquisition time delay between slices. Then, realignment was performed to correct the motion between time points. Head motion parameters were computed by estimating the translation in each direction and the angular rotation on each axis for each volume. All participant’s data were within the defined motion thresholds (i.e., translational or rotational motion parameters less than 2 mm or 2°). We also calculated frame-wise displacement (FD), which indexes the volume-to-volume changes in head position. Several nuisance covariates (the linear drift, the estimated motion parameters based on the Friston-24 model, the spike volumes with FD > 0.5 mm, the global signal, the white matter signal, and the cerebrospinal fluid signal) were regressed out from the data. Because global signal regression can enhance the detection of system-specific correlations and improve the correspondence to anatomical connectivity (Murphy & Fox, 2017), we included this step in the preprocessing of resting-state fMRI data. Then, the datasets were band-pass filtered using a frequency range of 0.01–0.1 Hz. In the normalization step, individual structural images were firstly co-registered with the mean functional images; the transformed structural images were then segmented and normalized to MNI space using a high-level nonlinear warping algorithm, that is, the diffeomorphic anatomical registration through exponentiated Lie algebra technique (Ashburner, 2007). Next, each filtered functional volume was spatially normalized to MNI space using the deformation parameters estimated during the above step and resampled into 3-mm isotropic voxel. Finally, all data were spatially smoothed with a Gaussian kernel of 6 × 6 × 6 mm^3^ full-width at half maximum (FWHM).

The HCP resting-state fMRI data were minimally preprocessed with echo planar imaging gradient distortion correction, motion correction, field bias correction, spatial transformation and normalization into MNI space, and artifact removal using independent component analysis (ICA) + FIX (Glasser et al., 2013, 2016). In addition to the HCP minimal preprocessing, several procedures were further performed using the DPABI software. Briefly, nuisance variables including the global, white matter, and cerebrospinal fluid signals were regressed out. Then, the data were band-pass filtered (0.01–0.1 Hz). Finally, the data were spatially smoothed with a 6-mm FWHM Gaussian kernel.

### Functional connectivity network mapping

We adopted the FCNM approach to construct PA hyper-activation and hypo-activation networks based on the extracted coordinates of the previously reported significant brain clusters with PA-induced hyper-activation (placebo > control contrast) and hypo-activation (placebo < control contrast), respectively (Figure 1). First, 4-mm radius spheres centered at each coordinate of a contrast were created and merged together to generate a contrast-specific combined seed mask (henceforth referred to as the contrast seed). Second, based on the preprocessed resting-state fMRI data, we computed a contrast seed-to-whole brain functional connectivity (FC) map for each subject, by calculating Pearson’s correlation coefficients between time courses of the contrast seed and each voxel within the whole brain, followed by Fisher’s *Z*-transformation to improve normality. Third, the subject-level FC maps were entered into a voxel-wise one-sample *t*-test to identify brain regions functionally connected to each contrast seed. Note that we only considered positive FC as the biological meaning of negative FC is still a matter of debate (Murphy et al., 2009; Murphy & Fox, 2017). Fourth, the resulting group-level *t* maps were thresholded at *P* < 0.05 corrected for multiple comparisons using a voxel-level family-wise error method and then binarized. Finally, the binarized maps were overlaid to produce two network probability maps, which were thresholded at 60% to yield the PA hyper-activation and hypo-activation networks, respectively.

### Relation to canonical brain networks

For ease of interpretability, we examined the spatial associations between the PA networks and eight well-established canonical brain networks. The seven cortical networks were defined as the visual, somatomotor, dorsal attention, ventral attention, limbic, frontoparietal, and default networks according to the Yeo et al. study (Yeo et al., 2011). The Human Brainnetome Atlas was adopted to define the subcortical network including the amygdala, hippocampus, basal ganglia, and thalamus (Fan et al., 2016). The proportion of overlapping voxels between each PA network and a canonical network to all voxels within the corresponding canonical network was computed to quantify their spatial association.

### Validation analyses

We conducted several validation analyses to test the robustness of our findings. First, we did the same analyses utilizing an independent validation dataset (i.e., the HCP) to evaluate the impact of dataset selection. Second, we repeated the FCNM procedure with use of 1-mm and 7-mm radius spheres to assess the effect of seed size. To quantify the spatial similarity between the PA networks from the main and validation analyses, we calculated a Dice coefficient, defined as 2 × (overlapping voxels) / [(network #1 voxels) + (network #2 voxels)]. Third, to examine the impact of overlap threshold selection, we repeated the FCNM procedure using 50% and 70% thresholds.

## Results

### Included studies

A total of 29 studies with 49 within-subject contrasts from 674 individuals were included in our analyses. Specifically, 25 studies with 25 placebo > control contrasts from 561 individuals were included in the PA hyper-activation analysis, and 22 studies with 24 placebo < control contrasts from 545 individuals were included in the PA hypo-activation analysis. Sample and imaging information of the included studies is summarized in Tables S3 and S4 in the Supplementary Materials.

### PA hyper-activation network

**Figure 3.**
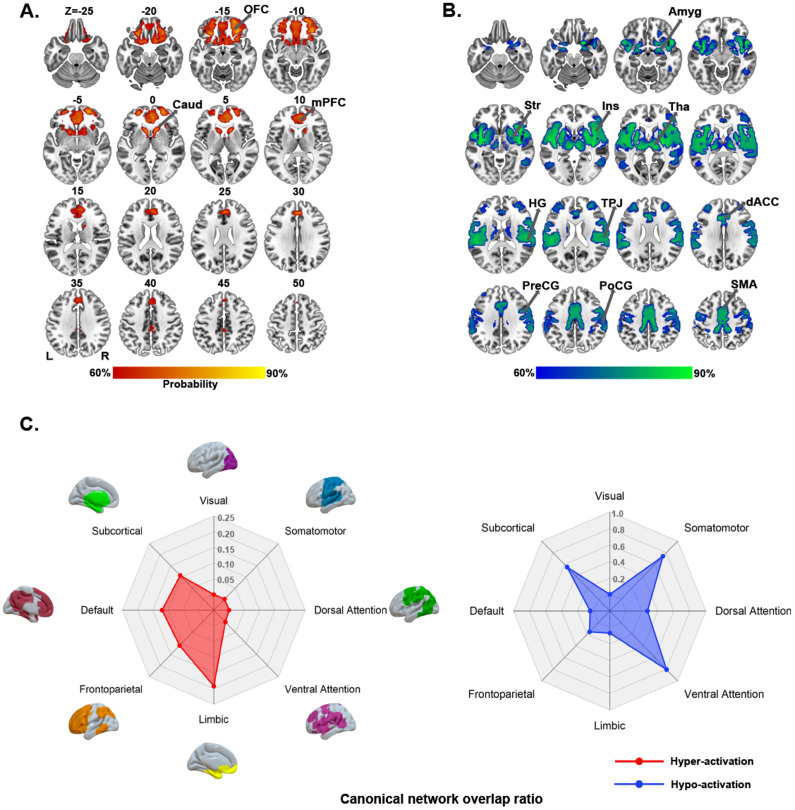
PA brain networks and their relation to canonical networks. Both PA hyper-activation (A) and PA hypo-activation (B) networks are presented as network probability maps thresholded at 60%, showing brain regions functionally connected to more than 60% of the contrast seeds. Polar plots (C) illustrate the proportion of overlapping voxels between each PA brain network (left panel: hyper-activation; right panel: hypo-activation) and a canonical network to all voxels within the corresponding canonical network. *Note*: Amyg, amygdala; Caud, caudate; dACC, dorsal anterior cingulate cortex; HG, Heschl’s gyrus; Ins, insula; L, left; mPFC, medial prefrontal cortex; OFC, orbitofrontal cortex; PA, placebo analgesia; PreCG, precentral gyrus; PoCG, postcentral gyrus; R, right; Str, striatum; SMA, supplementary motor area; Tha, thalamus; TPJ, temporoparietal junction.

The PA hyper-activation network manifested as a pattern of circumscribed brain regions, principally including the bilateral orbitofrontal cortex, medial prefrontal cortex, and caudate (Figure 3A). Regarding canonical brain networks, the PA hyper-activation network mainly involved the limbic (overlapping proportion: 19.20%), default (12.02%), and frontoparietal (10.92%) networks (Figure 3C, left panel).

### PA hypo-activation network

The PA hypo-activation network comprised a broadly distributed set of brain regions predominantly including the bilateral insula and operculum, dorsal anterior cingulate cortex, temporoparietal conjunction, precentral gyrus, postcentral gyrus, supplementary motor area, middle cingulate cortex, superior temporal gyrus, Heschl’s gyrus, striatum, thalamus, and amygdala (Figure 3B). With respect to canonical brain networks, the PA hypo-activation network primarily implicated the ventral attention (overlapping proportion: 80.45%), somatomotor (73.94%), and subcortical (55.42%) networks (Figure 3C, right panel).

### Validation analyses

The core regions of the PA networks derived from the discovery AMUD dataset were largely preserved when analyzing the validation HCP dataset (AMUD vs. HCP Dice coefficients: 0.567, 0.828) (Figure S1 in the Supplementary Materials). When repeating the FCNM procedure using 1-mm and 7-mm radius spheres, we found that the resultant PA networks were nearly identical to those using the 4-mm radius sphere (4-mm vs. 1-mm Dice coefficients: 0.941, 0.986; 4-mm vs. 7-mm Dice coefficients: 0.912, 0.980) (Figures S2 and S3 in the Supplementary Materials). Using the 50% and 70% thresholds yielded PA hyper-activation and hypo-activation networks that had larger and smaller spatial extents than those based on the 60% threshold (Figures S4 and S5 in the Supplementary Materials). However, the core areas of the PA hyper-activation and hypo-activation networks were still present across these thresholds. These results suggested the robustness of our findings to different datasets and methodological variation. For completeness, we constructed the PA brain networks based on negative FC and found that the hyper-activation network primarily overlapped with the visual (73.70%), somatomotor (35.46%), and dorsal attention (13.88%) networks, whereas the hypo-activation network mainly involved the default (65.57%), frontoparietal (45.21%), and visual (31.49%) networks (Figure S6 in the Supplementary Materials).

## Discussion

By integrating the novel FCNM framework and large-scale resting-state fMRI datasets, we reported the first study to examine network localization of brain functional alterations related to PA. Our analyses mapped the PA-induced hyper-activation and hypo-activation regions, which were highly neuroanatomically heterogeneous, to two different and specific brain networks. The PA hyper-activation network manifested as a pattern of circumscribed brain regions mainly involving the limbic, default, and frontoparietal networks. By contrast, the PA hypo-activation network comprised a broadly distributed set of brain regions primarily implicating the ventral attention, somatomotor, and subcortical networks. Aside from unifying the seemingly irreproducible results across previous neuroimaging studies on PA, the current findings may help refine our understanding of the brain network representations of PA, potentially contributing to elucidating its action mechanisms and providing a neural framework that may inform future clinical translation.

Prior neuroimaging studies and meta-analyses have identified brain correlates of PA in human experimental pain (Amanzio et al., 2013; Atlas & Wager, 2014; Fu et al., 2021; Zunhammer et al., 2021), reporting key regions such as the anterior cingulate cortex, anterior insula, amygdala, thalamus, periaqueductal gray, striatum, and posterior cingulate/precuneus. However, inconsistencies in the precise location and nature of these reported effects make it challenging to unify this research. Several possible explanations for the inconsistencies have been proposed, including small sample sizes, inherent noisiness of functional neuroimaging data, flexible study designs, variable analytical strategies, and rather liberal statistical inferences. In addition to these common concerns in neuroimaging research, earlier efforts have focused principally on regional brain effects, violating the major concept in modern neuroscience that neural processes in relation to a brain disorder or in response to a treatment do not act in isolation but rather rely on distributed, interconnected networks (Fornito et al., 2015). With regard to therapeutic interventions for brain disorders, indeed, there has been increasing interest in using updated network-based methodologies to localize the treatment effects to specific brain networks (Fox et al., 2014; Siddiqi et al., 2020). In the present study, we employed the FCNM approach to map the neuroanatomically heterogeneous brain functional alterations induced by PA to common networks, adding to the extant knowledge regarding network-level brain representations of placebo effects. Indeed, our results show that the consistent regional brain functional alterations induced by PA reported in the prior meta-analyses are largely localized within the PA networks we identified.

Specifically, the prefrontal cortex and anterior cingulate cortex, which are consistently reported in the meta-analyses, are located within the PA hyper-activation network, which manifested as a pattern of circumscribed brain regions mainly involving the limbic, default, and frontoparietal networks. Studies have shown that the prefrontal and cingulate cortex in the limbic system are of importance in emotion processing and pain regulation (Wager & Atlas, 2015). For example, the cingulate cortex was found to be particularly active in anticipatory and nociceptive processing, strongly associated with pain relief in placebo effects (Mogil, 2024). Meanwhile, the amygdala is also involved in mediating pain-related emotional experiences (Ji & Neugebauer, 2019), and its synergistic effect with the limbic regions is critical for placebo effects. The default network supports a broad category of processes related to affective appraisal. It is associated with spontaneous thought (Mason et al., 2007), autobiographical memory retrieval (Vincent et al., 2006), prospection (Buckner & Carroll, 2007), generating negative and positive emotion (Lindquist et al., 2012), assessing others’ mental states (Frith & Frith, 2006), and self-related processing (Gusnard, Akbudak, Shulman, & Raichle, 2001). The medial prefrontal activity overlaps with activity related to instructed fear, in which anxiety is generated by conceptual knowledge about associations between cues and shocks, without reinforcement (Mechias, Etkin, & Kalisch, 2010). Self-generated positive emotion consistently activates an overlapping, but more restricted, set of regions, including all the major elements of the mesolimbic dopamine system, including the ventromedial prefrontal cortex, striatum, and ventral tegmental area. There is a large body of literature suggesting that the frontoparietal network is implicated in guiding decisions and performance adjustment (Mason et al., 2007; Molinari et al., 2013). Research indicates that the frontoparietal network may integrate information from the external environment with stored internal representations (Miller, 2000), control top-down attention during conflict processing of alternative responses (Corbetta & Shulman, 2002), and control monitor conflict with subsequent adjustment in performance (Carter & van Veen, 2007; Crone, Wendelken, Donohue, & Bunge, 2006; Ridderinkhof, Ullsperger, Crone, & Nieuwenhuis, 2004). In an earlier study (O’Connor, Han, & Dobbins, 2010), investigators manipulated the correspondence between anticipated and actual recognition evidence by presenting valid or invalid anticipatory cues before recognition judgments. They found a reliable correlation between participants’ response biases and activation in the frontoparietal network, implying that a mechanism of expectation and conflict regulation may be engaged in cue modulatory effects on pain. Wager and colleagues found that increased anticipatory activity in the frontoparietal control network could predict placebo responses (Wager et al., 2011). Taken together, there is strong evidence that the frontoparietal network plays a key role in expectancy-induced modulation of pain.

Our PA hypo-activation network includes the key ‘pain matrix’ regions that were consistently identified to exhibit reduced activity during PA in a recent meta-analysis (Zunhammer et al., 2021), such as the anterior/posterior insular cortex, thalamus, and secondary somatosensory cortex. The PA hypo-activation network comprised a broadly distributed set of brain regions primarily implicating the ventral attention, somatomotor, and subcortical networks. The ventral attention network is typically implicated in stimulus-driven attentional control (Ridderinkhof et al., 2004). Li et al. explored functional connectivity related to pain stimulation at the whole brain level and found that pain stimulation was related to enhancement of functional connectivity within and between multiple brain networks including the ventral attention network (Li et al., 2022). This invites the speculation that enhanced connectivity of the ventral attention network may constitute the neural basis of pain salience. As for the somatomotor network, Wagner et al. found that after pain stimulation, the connection between the somatosensory network and other networks in the PA group was weakened compared with the control group, suggesting that placebo manipulation may produce analgesic effect by separating the somatosensory network of pain processing and the descending regulatory network of pain (Wagner et al., 2020). With respect to the subcortical network, placebo analgesia is associated with changes in fronto-striatal-brainstem circuits, including prefrontal suppression of striatal prediction errors (Schenk et al., 2017; Wager & Atlas, 2015). This top-down signal converges on the periaqueductal gray-rostroventromedial medulla (PAG-RVM) axis, a final common pathway for both opioid and placebo analgesia (Chen et al., 2024; Livrizzi et al., 2025). Causal animal studies confirm that silencing ventrolateral PAG → RVM neurons or their cortical inputs from medial prefrontal cortex/anterior cingulate cortex (ACC) abolishes conditioned placebo analgesia (Livrizzi et al., 2025). This core descending pathway is embedded in a broader modulatory network. For example, attentional analgesia recruits not only the ACC-PAG-RVM pathway but also a bidirectional ACC-locus coeruleus loop, indicating noradrenergic involvement (Oliva et al., 2021). The parabrachial nucleus (PB), a hub for affective-motivational pain processing, shows reduced activity during placebo. Through its connections with the amygdala and the PAG-RVM axis, the PB helps translate forebrain expectations into tuned descending inhibition (Crawford et al., 2021; Yanes & Akintola, 2022). Another plausible interpretation is that placebo-associated down-regulation seems to affect thalamocortical pathways that are involved in nociception and pain (Duerden & Albanese, 2013; Segerdahl et al., 2015).

There are several limitations to the present study. First, as our findings are largely based on the prior studies implementing experimental placebo interventions in healthy volunteers, they may not generalize to clinical populations. In addition, we utilized resting-state fMRI data from an independent sample of healthy adults to carry out the network localization analysis. Theoretically, it appears preferable to use fMRI data from a sample with demographic and clinical characteristics comparable to those of the participants in the selected studies. Nevertheless, there is a growing body of evidence indicating that sample selection has negligible influence on network localization results (Boes et al., 2015; Fox et al., 2014; Horn et al., 2017). Second, the included studies covered a wide range of experimental placebo paradigms and conditions. This appears favorable in terms of establishing the broad generalizability of results, but it also means that the results have to generalize over many sources of variation: paradigm, sample, scanner, and choice of analysis methods. Effect size estimates are thus likely overly conservative compared with what may be possible as analysis methods continue to become standardized and methodological advances reduce inter-study variability. Third, a relatively liberal overlapping threshold of 60% was utilized for network localization, due to the fact that several potential confounders may hamper the possibility to find a common brain network at a more stringent threshold. Fourth, our study identifies co-activation or co-connectivity patterns associated with PA but cannot determine the causal or hierarchical role of the networks or their constituent nodes. Finally, the substantial heterogeneity in the included studies, such as differences in the number of foci, sample size, and effect size, may introduce a potential ‘over-representation’ bias. As no consensus has been reached on how to evaluate and adjust for the effects of these variations on network localization, further research would benefit from future improvement in analytic approaches, for example, developing a more robust, weighted approach or selecting one random coordinate per contrast for validation analysis.

In conclusion, we combined the novel FCNM framework and large-scale resting-state fMRI datasets to map the neuroanatomically heterogeneous regions with PA-induced hyper-activation and hypo-activation to two distinct and specific brain networks. Our work may inform the theoretical and clinical understanding of PA. For one, by reconciling the inconsistency across studies, network localization may provide a generic unifying framework that holds value in mitigating concerns around the reproducibility of neuroimaging findings related to PA. For another, our findings regarding the brain network representations of PA may contribute to a deeper understanding of its action mechanisms and provide a neural framework that may inform future clinical translation.

## Supporting information

10.1017/S0033291726103924.sm001 Zhang et al. supplementary material Zhang et al. supplementary material

## Data Availability

The data and analysis codes used in the preparation of this article are publicly available at https://osf.io/3vxrj/.

## Supplementary material

The supplementary material for this article can be found at http://doi.org/10.1017/S0033291726103924.
