# Supplementary material for: Brain network representations of placebo analgesia
Source: Psychol Med. 2026 May 5;56:e131. doi: 10.1017/S0033291726103924 (PMC13161808; doi:10.1017/S0033291726103924)
Supplement: Zhang et al. supplementary material [file S0033291726103924sup001.docx]

**Supplementary materials**

**Table S1.** Demographic information of the discovery and validation datasets

| **Dataset** | **Sample size** | **Age (years)** | **Gender (F/M)** |
| --- | --- | --- | --- |
| AMUD | 1113 | 32.66 ± 12.78 | 643/470 |
| HCP | 1093 | 28.78 ± 3.69 | 594/499 |

Age is expressed as mean ± standard deviation. Abbreviations: AMUD, Anhui Medical University Dataset; F, female; HCP, Human Connectome Project; M, male.

**Table S2.** Resting-state fMRI parameters of the discovery and validation datasets

| **Parameter** | **AMUD** | **HCP** |
| --- | --- | --- |
| Scanner | 3.0T General Electric Discovery MR750w | 3.0T Siemens Skyra |
| Sequence | GRE-SS-EPI | GRE-EPI |
| TR (ms) | 2000 | 720 |
| TE (ms) | 30 | 33.1 |
| FA (°) | 90 | 52 |
| FOV (mm^2^) | 220 × 220 | 208 × 180 |
| Matrix size | 64 × 64 | 104 × 90 |
| Slice thickness (mm) | 3 | 2 |
| Slice gap (mm) | 1 | 0 |
| Slices | 35 | 72 |
| Time points | 185 | 1200 |

Abbreviations: AMUD, Anhui Medical University Dataset; EPI, echo planar imaging; FA, flip angle; fMRI, functional magnetic resonance imaging; FOV, field of view; GRE, gradient echo; HCP, Human Connectome Project; SS, single shot; TE, echo time; TR, repetition time.

**Table S3.** Summary of studies included for the PA hyper-activation analysis (placebo > control, n = 25)

| **Study** | **N (male)** | **Mean age (SD)** | | **Age range** | **Type of pain** | **Type of manipulation** | **Methodology** | **Pain stage** | **Soft** | **Tesla** |
| --- | --- | --- | --- | --- | --- | --- | --- | --- | --- | --- |
| Atlas et al. (Atlas et al., 2012) (2012) | 19 (9) | 24.4 (N/A) | N/A | | Thermal | Verbal instruction | fMRI | 2 | SPM5 | 1.5 |
| Bingel et al. (Bingel, Lorenz, Schoell, Weiller, & Buchel, 2006) (2006) | 19 (15) | 24.0 (N/A) | 18–32 | | Laser | Conditioning | fMRI | 2 | SPM2 | 1.5 |
| Crawford et al. (Crawford et al., 2023) (2023) | 38 (20) | 25 (0.8) | 20–37 | | Thermal | Conditioning | fMRI | 2 | SPM12 | 7 |
| Eippert et al. (Eippert et al., 2009) (2009) | 48 (N/A) | 26.13 (N/A) | 20–40 | | Thermal | Verbal instruction | fMRI | 2 | SPM5 | N/A |
| Ellingsen et al. (Ellingsen et al., 2013) (2013) | 28 (N/A) | N/A | N/A | | Mechanical | Verbal instruction | fMRI | 2 | FEAT | 3 |
| Fehse et al. (Fehse, Maikowski, Simmank, Gutyrchik, & Meissner, 2015) (2015) | 30 (30) | 32.0 (6.39) | N/A | | Thermal | Verbal instruction | fMRI | 2 | SPM8 | 3 |
| Geuter et al. (Geuter, Eippert, Hindi Attar, & Büchel, 2013) (2013) | 40 (40) | 26.0 (N/A) | 19–40 | | Thermal | Conditioning | fMRI | 1, 2 | SPM8 | 3 |
| Jensen et al. (Jensen et al., 2015) (2015) | 24 (14) | 25.0 (5.0) | N/A | | Thermal | Conditioning | fMRI | 2 | SPM8 | 3 |
| Kong et al. (Kong et al., 2006) (2006) | 16 (9) | N/A | N/A | | Thermal | Conditioning | fMRI | 2 | SPM2 | 3 |
| Kong et al. (Kong et al., 2009) (2009) | 12 (6) | N/A | N/A | | Thermal | Conditioning | fMRI | 2 | SPM2 | 3 |
| Lu et al. (Lu et al., 2010) (2010) | 14 (5) | 23.9 (3.9) | N/A | | Visceral (esophageal) | Conditioning | fMRI | 1, 2 | SPM5 | 3 |
| Lui et al. (Lui et al., 2010) (2010) | 31 (13) | 23.5 (N/A) | N/A | | Laser | Conditioning | fMRI | 1, 2 | SPM5 | 3 |
| Nemoto et al. (Nemoto, Nemoto, Toda, Mikuni, & Fukuyama, 2007) (2007) | 5 (N/A) | N/A | 21–25 | | Thermal | Conditioning | PET (rCBF) | 3 | SPM99 | N/A |
| Schmid et al. (Schmid et al., 2015) (2015) | 17 (2) | 38.29 (3.38) | N/A | | Visceral  (rectal) | Verbal instruction | fMRI | 1, 2 | SPM8 | 3 |
| Theysohn et al. (Theysohn et al., 2014) (2014) | 15 (0) | N/A | N/A | | Visceral (rectal) | Verbal instruction | fMRI | 1, 2 | SPM8 | 3 |
| van der Meulen et al. (van der Meulen, Kamping, & Anton, 2017) (2017) | 30 (13) | 25.7 (5.5) | N/A | | Thermal | Verbal instruction | fMRI | 1, 2 | SPM8 | N/A |
| Wager et al. (Wager, Scott, & Zubieta, 2007) (2007) | 15 (N/A) | N/A | 20–30 | | Thermal | Conditioning | PET | 1, 2 | SPM2 | N/A |
| Wager et al. (Wager et al., 2004) (2004) | 23 (N/A) | N/A | N/A | | Thermal | Verbal instruction | fMRI | 1, 2 | SPM99 | 3 |
| Watson et al. (Watson et al., 2009) (2009) | 11 (5) | N/A | 19–36 | | Electrical | Verbal instruction | fMRI | 1, 2 | FLIRT | 3 |
| Wrobel et al. (Wrobel, Wiech, Forkmann, Ritter, & Bingel, 2014) (2014) | 29 (N/A) | N/A | N/A | | Thermal | Conditioning | fMRI | 2 | SPM8 | 3 |
| Zeidan et al. (Zeidan, Emerson, et al., 2015) (2015) | 19 (10) | 27.42 (5.22) | N/A | | Thermal | Conditioning | fMRI | 2 | FSL | 3 |
| Zeidan et al. (Zeidan, Lobanov, Kraft, & Coghill, 2015) (2015) | 15 (8) | 26.0 (N/A) | 23–30 | | Thermal | Conditioning | fMRI | 2 | FSL | 1.5 |
| Zhang et al. (Zhang, Qin, Guo, & Luo, 2011) (2011) | 13 (1) | N/A | N/A | | Laser | Conditioning | fMRI | 2 | SPM5 | 3 |
| Zhang et al. (Zhang, Guo, Zhang, & Luo, 2013) (2013) | 26 (2) | N/A | N/A | | Laser | Conditioning | fMRI | 2 | SPM5 | 3 |
| Zhao et al. (Zhao, Liu, Zhang, Luo, & Zhang, 2020) (2020) | 24 (6) | 22.88 (1.96) | N/A | | Laser | Conditioning | fMRI | 2 | SPM8 | 3 |

Abbreviations: fMRI, functional magnetic resonance imaging; N/A, not available; PET, positron emission tomography; pain stage 1, pre-stimulation pain expectation; pain stage 2, start and end of the pain stimuli administration; pain stage 3, subjective pain intensity rating; rCBF, regional cerebral blood flow; SD, standard deviation.

**Table S4.** Summary of studies included for the PA hypo-activation analysis (placebo < control, n = 22)

| **Study** | **N (male)** | **Mean age (SD)** | **Age range** | **Type of pain** | **Type of manipulation** | **Methodology** | **Pain stage** | **Soft** | **Tesla** |
| --- | --- | --- | --- | --- | --- | --- | --- | --- | --- |
| Atlas et al. (Atlas et al., 2012) (2012) | 19 (9) | 24.4 (N/A) | N/A | Thermal | Verbal instruction | fMRI | 2 | SPM5 | 1.5 |
| Crawford et al. (Crawford et al., 2023) (2023) | 38 (20) | 25.0 (0.8) | 20–37 | Thermal | Conditioning | fMRI | 2 | SPM12 | 7 |
| Eippert et al. (Eippert et al., 2009) (2009) | 48 (N/A) | 26.13 (N/A) | 20–40 | Thermal | Verbal instruction | fMRI | 2 | SPM5 | N/A |
| Elsenbruch et al. (Elsenbruch et al., 2012) (2012) | 18 (N/A) | N/A | N/A | Visceral (rectal) | Verbal instruction | fMRI | 1, 2 | SPM5 | 1.5 |
| Geuter et al. (Geuter et al., 2013) (2013) | 40 (40) | 26.0 (N/A) | 19–40 | Thermal | Conditioning | fMRI | 1, 2 | SPM8 | 3 |
| Kessner et al. (Mouraux et al., 2014) (2014) | 39 (N/A) | 26 (N/A) | 22–36 | Thermal | Conditioning | fMRI | 2 | SPM8 | 3 |
| Kong et al. (Kong et al., 2006) (2006) | 16 (9) | N/A | N/A | Thermal | Conditioning | fMRI | 2 | SPM2 | 3 |
| Kong et al. (Kong et al., 2009) (2009) | 12 (6) | N/A | N/A | Thermal | Conditioning | fMRI | 2 | SPM2 | 3 |
| Lu et al. (Lu et al., 2010) (2010) | 14 (5) | 23.9 (3.9) | N/A | Visceral (esophageal) | Conditioning | fMRI | 1, 2 | SPM5 | 3 |
| Nemoto et al. (Nemoto et al., 2007) (2007) | 5 (N/A) | N/A | 21–25 | Thermal | Conditioning | PET (rCBF) | 3 | SPM99 | N/A |
| Schenk et al. (Schenk, Sprenger, Geuter, & Büchel, 2014) (2014) | 32 (17) | 25.6 (3.3) | 19–33 | Visceral (rectal) | Verbal instruction | fMRI | 1 | SPM8 | 3 |
| Schmid et al. (Schmid et al., 2015) (2015) | 17 (2) | 38.29 (3.38) | N/A | Visceral (rectal) | Verbal instruction | fMRI | 2 | SPM8 | 3 |
| Theysohn et al. (Theysohn et al., 2014) (2014) | 15 (0) | N/A | N/A | Visceral (rectal) | Verbal instruction | fMRI | 2 | SPM8 | 3 |
| Theysohn et al. (Theysohn et al., 2014) (2014) | 15 (15) | N/A | N/A | Visceral (rectal) | Verbal instruction | fMRI | 2 | SPM8 | 3 |
| van der Meulen et al. (van der Meulen et al., 2017) (2017) | 30 (13) | 25.7 (5.5) | N/A | Thermal | Verbal instruction | fMRI | 1, 2 | SPM8 | N/A |
| Wager et al. (Wager et al., 2007) (2007) | 15 (N/A) | N/A | 20–30 | Thermal | Conditioning | PET | 1, 2 | SPM2 | N/A |
| Wager et al. (Wager et al., 2004) (2004) | 24 (N/A) | N/A | N/A | Laser | Verbal instruction | fMRI | 2 | SPM99 | 3 |
| Wager et al. (Wager et al., 2004) (2004) | 23 (N/A) | N/A | N/A | Thermal | Verbal instruction | fMRI | 1, 2 | SPM99 | 3 |
| Wrobel et al. (Wrobel et al., 2014) (2014) | 29 (N/A) | N/A | N/A | Thermal | Conditioning | fMRI | 2 | SPM8 | 3 |
| Yang et al. (Yang et al., 2021) (2021) | 23 (0) | 20.22 (1.38) | 20–25 | orthodontic | Verbal instruction | fMRI | 2 | FEAT | 3 |
| Zeidan et al. (Zeidan, Emerson, et al., 2015) (2015) | 19 (10) | 27.42 (5.22) | N/A | Thermal | Conditioning | fMRI | 2 | FSL | 3 |
| Zeidan et al. (Zeidan, Lobanov, et al., 2015) (2015) | 15 (8) | 26.0 (N/A) | 23–30 | Thermal | Conditioning | fMRI | 2 | FSL | 1.5 |
| Zhang et al. (Zhang et al., 2011) (2011) | 13 (1) | N/A | N/A | Laser | Conditioning | fMRI | 2 | SPM5 | 3 |
| Zhang et al. (Zhang et al., 2013) (2013) | 26 (2) | N/A | N/A | Laser | Conditioning | fMRI | 2 | SPM5 | 3 |

Abbreviations: fMRI, functional magnetic resonance imaging; N/A, not available; PET, positron emission tomography; pain stage 1, pre-stimulation pain expectation; pain stage 2, start and end of the pain stimuli administration; pain stage 3, subjective pain intensity rating; rCBF, regional cerebral blood flow; SD, standard deviation.


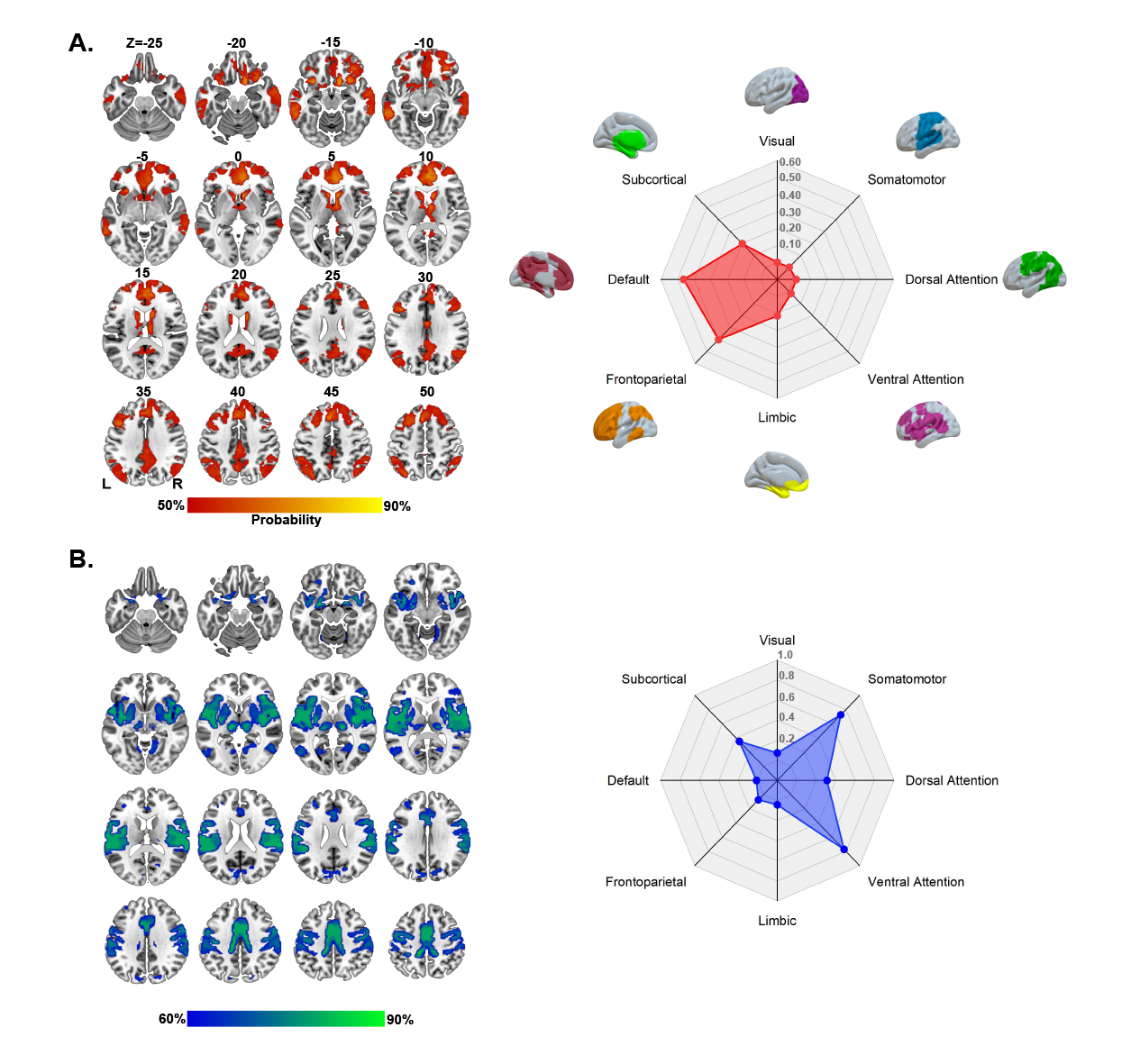


**Figure S1.** PA brain networks derived from the HCP dataset. **A.** PA hyper-activation network (left panel) and its relation to canonical brain networks (right panel). **B.** PA hypo-activation network (left panel) and its relation to canonical brain networks (right panel). Polar plots illustrate the proportion of overlapping voxels between each PA brain network and a canonical network to all voxels within the corresponding canonical network. Abbreviation: HCP, Human Connectome Project; L, left; PA, placebo analgesia; R, right.


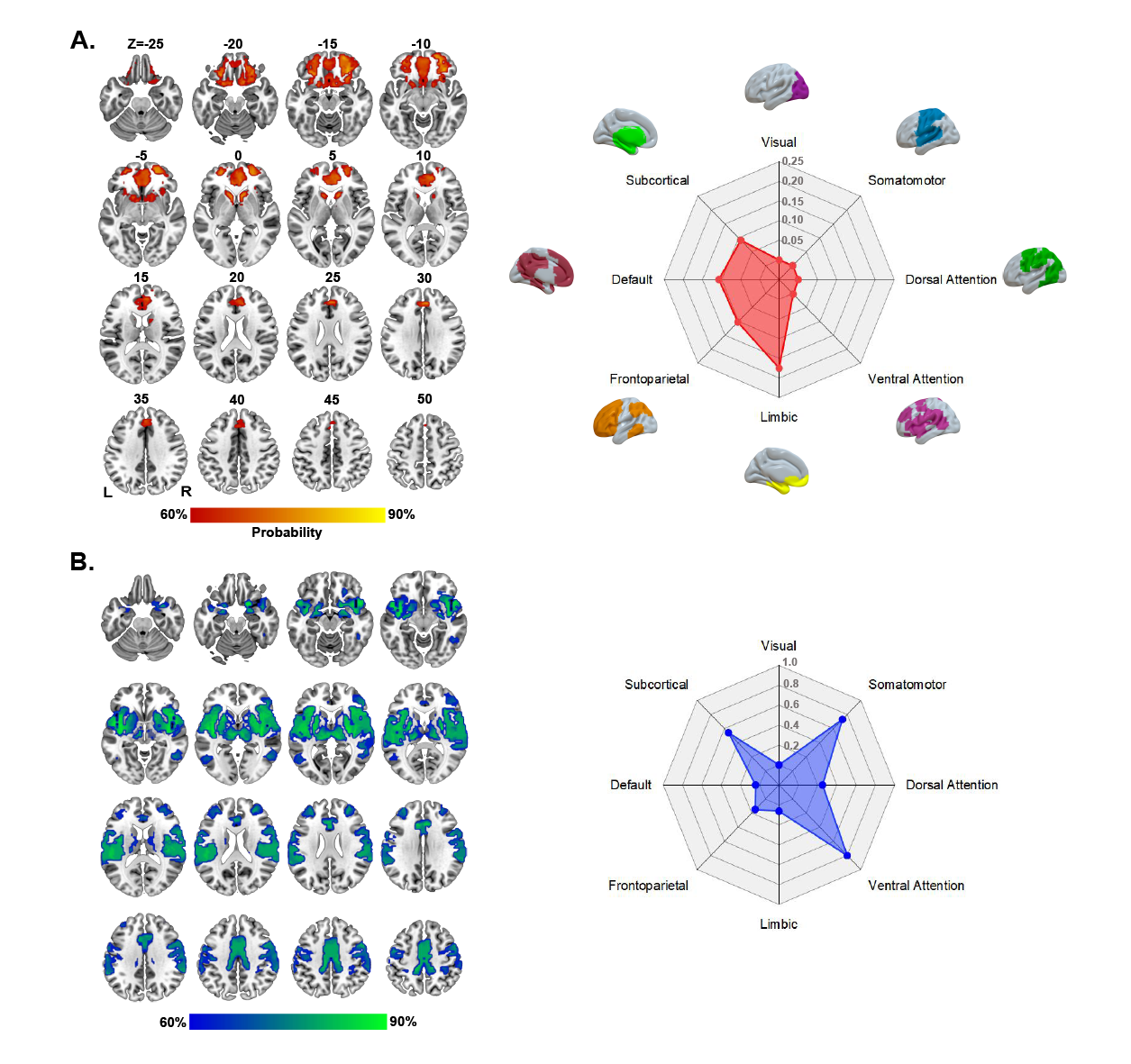


**Figure S2.** PA brain networks based on 1-mm radius sphere. **A.** PA hyper-activation network (left panel) and its relation to canonical brain networks (right panel). **B.** PA hypo-activation network (left panel) and its relation to canonical brain networks (right panel). Polar plots illustrate the proportion of overlapping voxels between each PA brain network and a canonical network to all voxels within the corresponding canonical network. Abbreviation: L, left; PA, placebo analgesia; R, right.


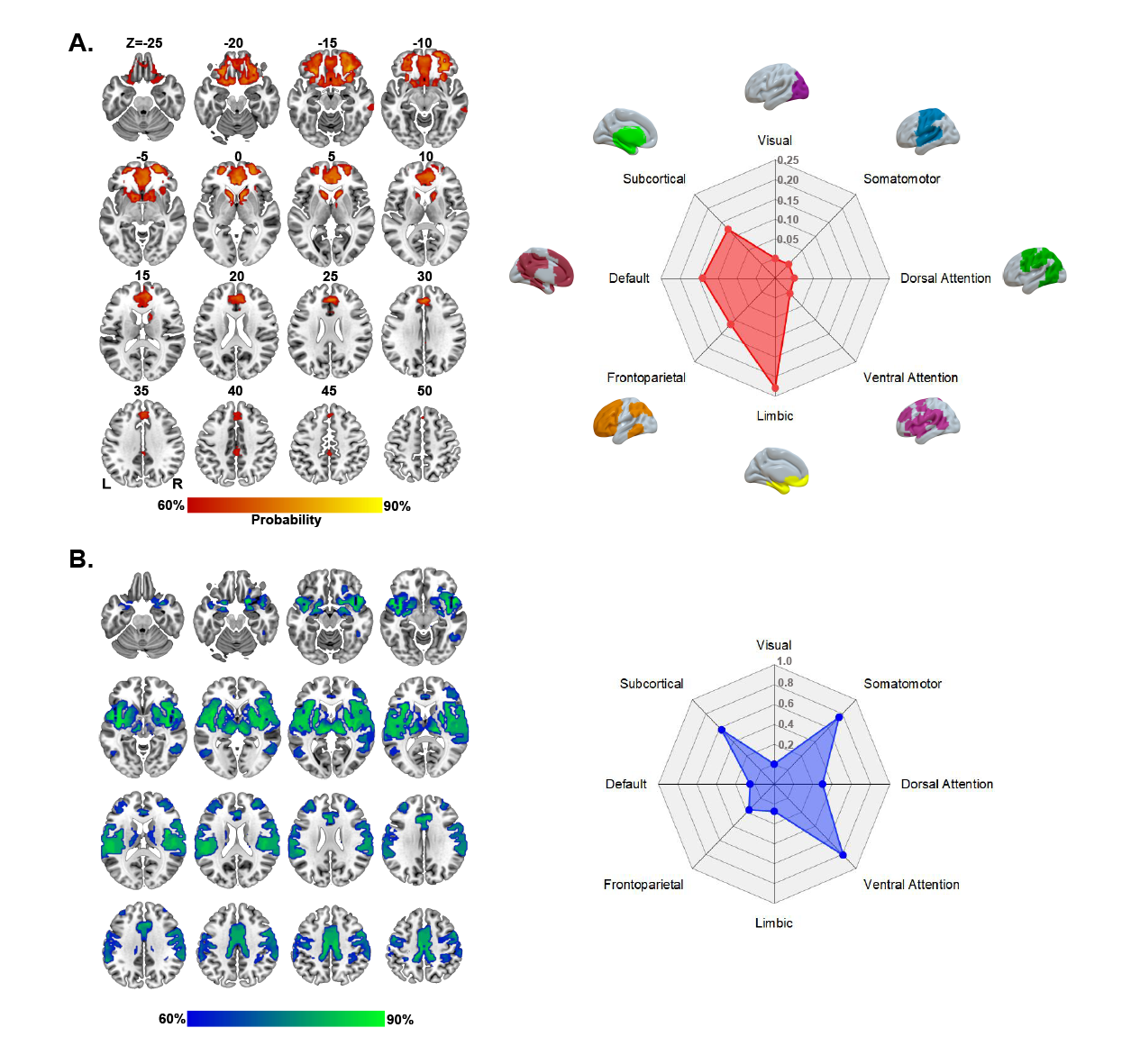


**Figure S3.** PA brain networks based on 7-mm radius sphere. **A.** PA hyper-activation network (left panel) and its relation to canonical brain networks (right panel). **B.** PA hypo-activation network (left panel) and its relation to canonical brain networks (right panel). Polar plots illustrate the proportion of overlapping voxels between each PA brain network and a canonical network to all voxels within the corresponding canonical network. Abbreviation: L, left; PA, placebo analgesia; R, right.

**Figure** **S4.** PA hyper-activation networks based on the 50% (A), 60% (B), and 70% (C) thresholds. Abbreviations: L, left; PA, placebo analgesia; R, right.


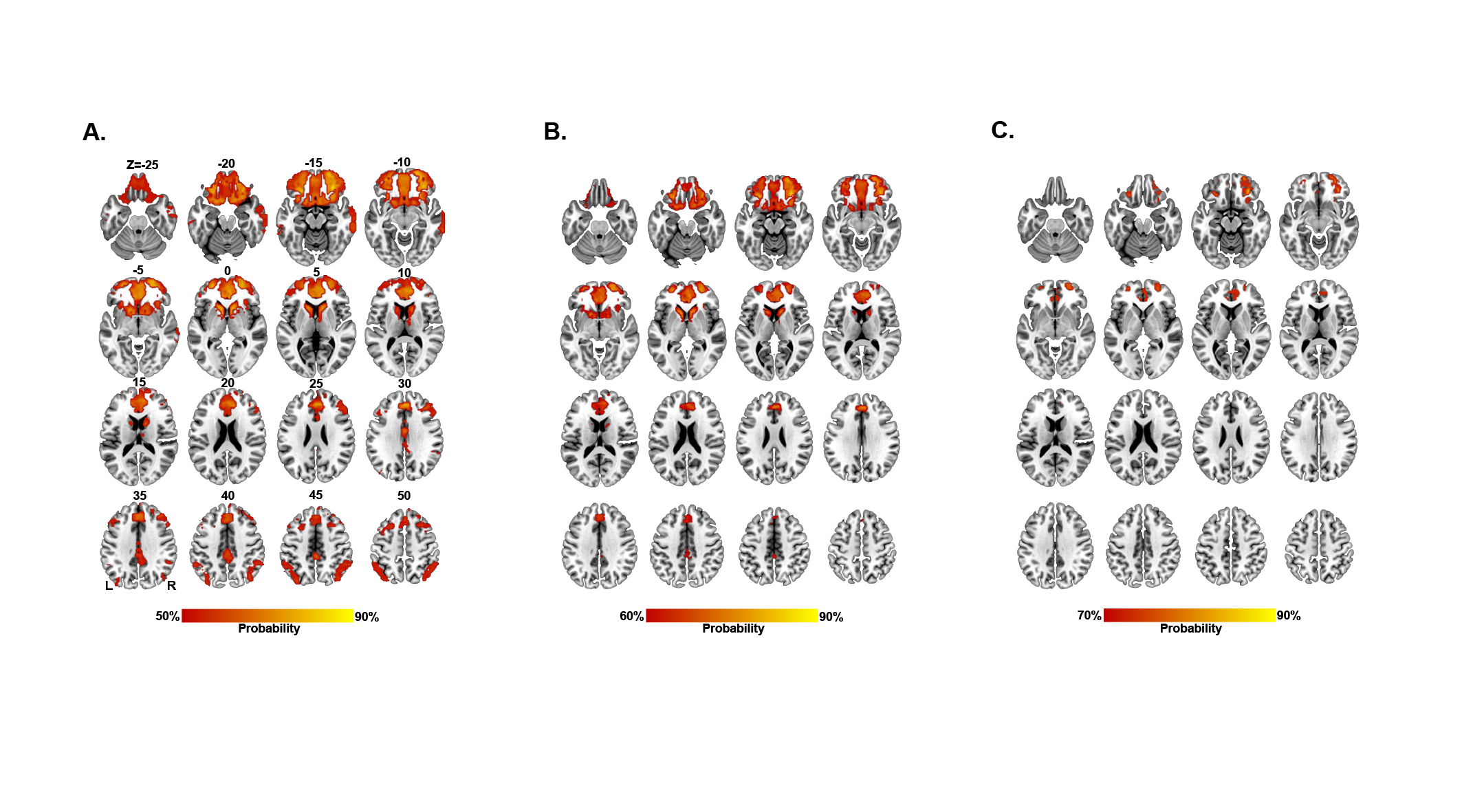


**Figure S5**. PA hypo-activation networks based on the 50% (A), 60% (B), and 70% (C) thresholds. Abbreviations: L, left; PA, placebo analgesia; R, right.


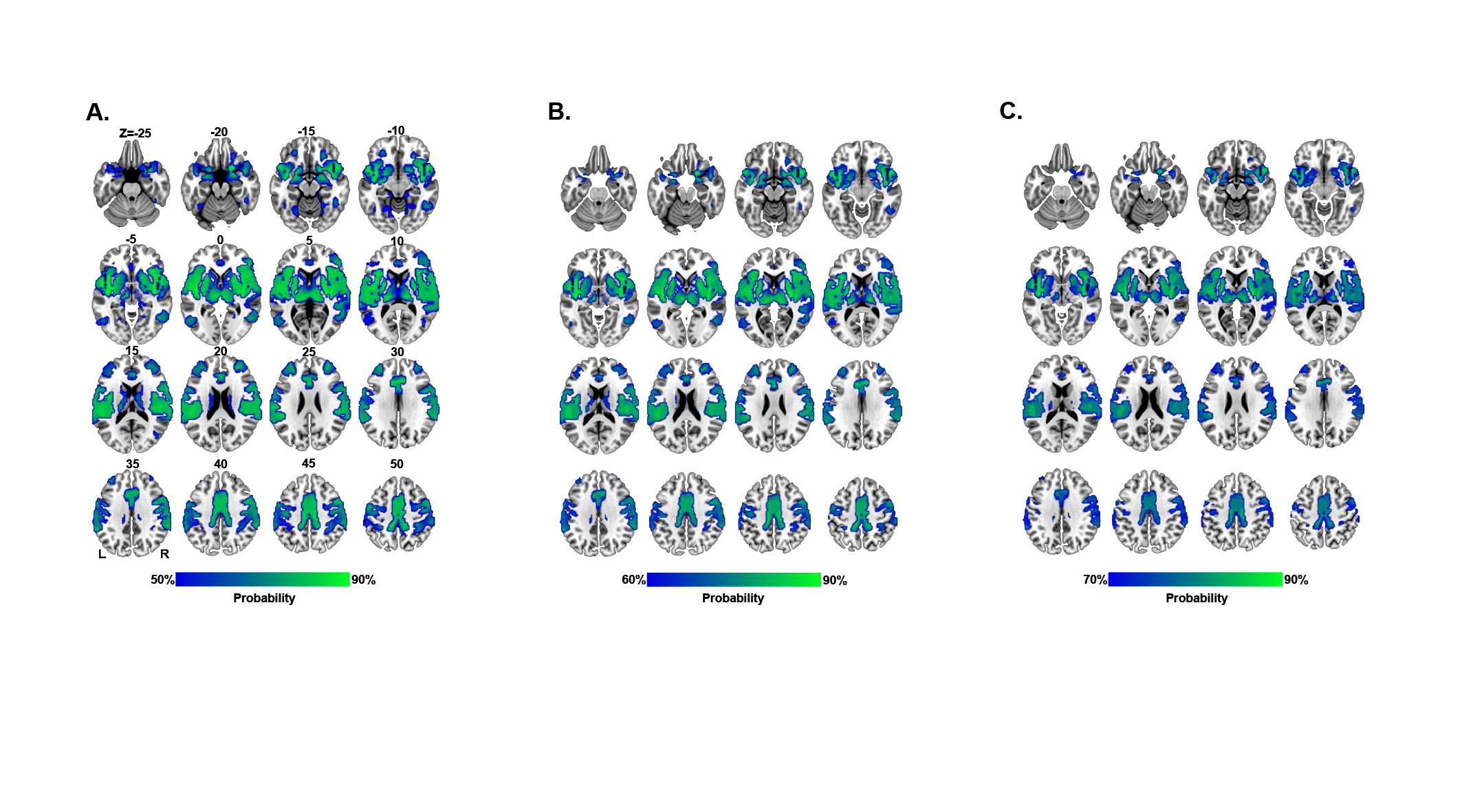

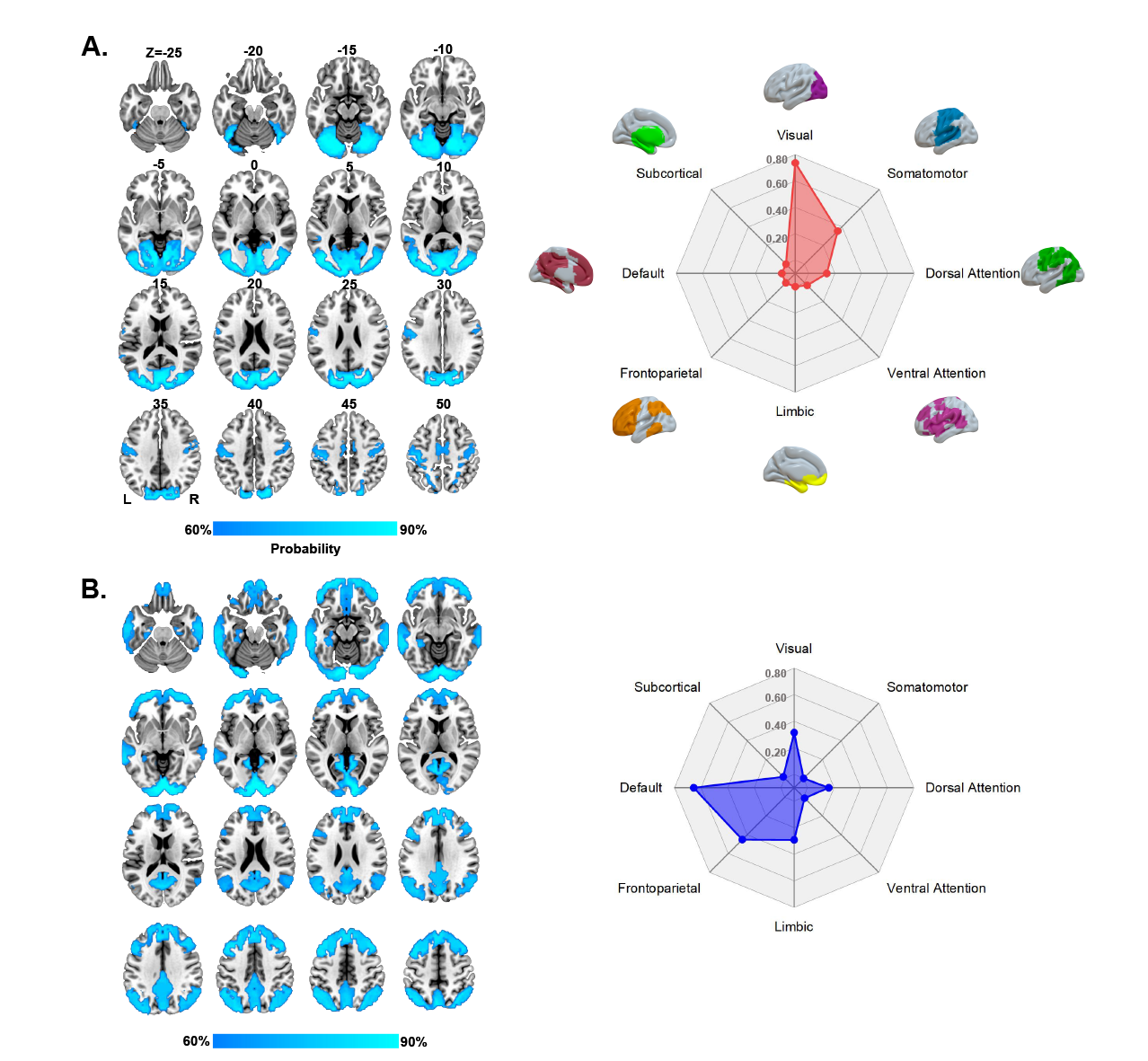


**Figure S6.** PA brain networks based on negative functional connectivity. **A.** PA hyper-activation network (left panel) and its relation to canonical brain networks (right panel). **B.** PA hypo-activation network (left panel) and its relation to canonical brain networks (right panel). Polar plots illustrate the proportion of overlapping voxels between each PA brain network and a canonical network to all voxels within the corresponding canonical network. Abbreviations: L, left; PA, placebo analgesia; R, right.

**References**

Atlas, L. Y., Whittington, R. A., Lindquist, M. A., Wielgosz, J., Sonty, N., & Wager, T. D. (2012). Dissociable Influences of Opiates and Expectations on Pain. *Journal of Neuroscience, 32*(23), 8053-8064. doi:10.1523/jneurosci.0383-12.2012

Bingel, U., Lorenz, J., Schoell, E., Weiller, C., & Buchel, C. (2006). Mechanisms of placebo analgesia: rACC recruitment of a subcortical antinociceptive network. *Pain, 120*(1-2), 8-15. doi:10.1016/j.pain.2005.08.027

Crawford, L. S., Mills, E. P., Peek, A., Macefield, V. G., Keay, K. A., & Henderson, L. A. (2023). Function and biochemistry of the dorsolateral prefrontal cortex during placebo analgesia: how the certainty of prior experiences shapes endogenous pain relief. *Cerebral Cortex, 33*(17), 9822-9834. doi:10.1093/cercor/bhad247

Eippert, F., Bingel, U., Schoell, E. D., Yacubian, J., Klinger, R., Lorenz, J., & Büchel, C. (2009). Activation of the Opioidergic Descending Pain Control System Underlies Placebo Analgesia. *Neuron, 63*(4), 533-543. doi:10.1016/j.neuron.2009.07.014

Ellingsen, D.-M., Wessberg, J., Eikemo, M., Liljencrantz, J., Endestad, T., Olausson, H., & Leknes, S. (2013). Placebo improves pleasure and pain through opposite modulation of sensory processing. *Proceedings of the National Academy of Sciences, 110*(44), 17993-17998. doi:10.1073/pnas.1305050110

Elsenbruch, S., Kotsis, V., Benson, S., Rosenberger, C., Reidick, D., Schedlowski, M., . . . Gizewski, E. R. (2012). Neural mechanisms mediating the effects of expectation in visceral placebo analgesia: An fMRI study in healthy placebo responders and nonresponders. *Pain, 153*(2), 382-390. doi:10.1016/j.pain.2011.10.036

Fehse, K., Maikowski, L., Simmank, F., Gutyrchik, E., & Meissner, K. (2015). Placebo Responses to Original vs. Generic ASA Brands During Exposure to Noxious Heat: A Pilot fMRI Study of Neurofunctional Correlates. *Pain Med, 16*(10), 1967-1974. doi:10.1111/pme.12783

Geuter, S., Eippert, F., Hindi Attar, C., & Büchel, C. (2013). Cortical and subcortical responses to high and low effective placebo treatments. *NeuroImage, 67*, 227-236. doi:10.1016/j.neuroimage.2012.11.029

Jensen, K. B., Kaptchuk, T. J., Chen, X., Kirsch, I., Ingvar, M., Gollub, R. L., & Kong, J. (2015). A Neural Mechanism for Nonconscious Activation of Conditioned Placebo and Nocebo Responses. *Cerebral Cortex, 25*(10), 3903-3910. doi:10.1093/cercor/bhu275

Kong, J., Gollub, R. L., Rosman, I. S., Webb, J. M., Vangel, M. G., Kirsch, I., & Kaptchuk, T. J. (2006). Brain Activity Associated with Expectancy-Enhanced Placebo Analgesia as Measured by Functional Magnetic Resonance Imaging. *The Journal of Neuroscience, 26*(2), 381-388. doi:10.1523/jneurosci.3556-05.2006

Kong, J., Kaptchuk, T. J., Polich, G., Kirsch, I., Vangel, M., Zyloney, C., . . . Gollub, R. (2009). Expectancy and treatment interactions: A dissociation between acupuncture analgesia and expectancy evoked placebo analgesia. *NeuroImage, 45*(3), 940-949. doi:10.1016/j.neuroimage.2008.12.025

Lu, H.-C., Hsieh, J.-C., Lu, C.-L., Niddam, D. M., Wu, Y.-T., Yeh, T.-C., . . . Lee, S.-D. (2010). Neuronal correlates in the modulation of placebo analgesia in experimentally-induced esophageal pain: A 3T-fMRI study. *Pain, 148*(1), 75-83. doi:10.1016/j.pain.2009.10.012

Lui, F., Colloca, L., Duzzi, D., Anchisi, D., Benedetti, F., & Porro, C. A. (2010). Neural bases of conditioned placebo analgesia. *Pain, 151*(3), 816-824. doi:10.1016/j.pain.2010.09.021

Mouraux, A., Kessner, S., Forkmann, K., Ritter, C., Wiech, K., Ploner, M., & Bingel, U. (2014). The Effect of Treatment History on Therapeutic Outcome: Psychological and Neurobiological Underpinnings. *PLoS ONE, 9*(10). doi:10.1371/journal.pone.0109014

Nemoto, H., Nemoto, Y., Toda, H., Mikuni, M., & Fukuyama, H. (2007). Placebo analgesia: a PET study. *Experimental Brain Research, 179*(4), 655-664. doi:10.1007/s00221-006-0821-z

Schenk, L. A., Sprenger, C., Geuter, S., & Büchel, C. (2014). Expectation requires treatment to boost pain relief: An fMRI study. *Pain, 155*(1), 150-157. doi:10.1016/j.pain.2013.09.024

Schmid, J., Langhorst, J., Gaß, F., Theysohn, N., Benson, S., Engler, H., . . . Elsenbruch, S. (2015). Placebo analgesia in patients with functional and organic abdominal pain: a fMRI study in IBS, UC and healthy volunteers. *Gut, 64*(3), 418-427. doi:10.1136/gutjnl-2013-306648

Theysohn, N., Schmid, J., Icenhour, A., Mewes, C., Forsting, M., Gizewski, E. R., . . . Benson, S. (2014). Are there sex differences in placebo analgesia during visceral pain processing? A fMRI study in healthy subjects. *Neurogastroenterology & Motility, 26*(12), 1743-1753. doi:10.1111/nmo.12454

van der Meulen, M., Kamping, S., & Anton, F. (2017). The role of cognitive reappraisal in placebo analgesia: an fMRI study. *Social Cognitive and Affective Neuroscience, 12*(7), 1128-1137. doi:10.1093/scan/nsx033

Wager, T. D., Rilling, J. K., Smith, E. E., Sokolik, A., Casey, K. L., Davidson, R. J., . . . Cohen, J. D. (2004). Placebo-induced changes in FMRI in the anticipation and experience of pain. *Science, 303*(5661), 1162-1167. doi:10.1126/science.1093065

Wager, T. D., Scott, D. J., & Zubieta, J. K. (2007). Placebo effects on human mu-opioid activity during pain. *Proc Natl Acad Sci U S A, 104*(26), 11056-11061. doi:10.1073/pnas.0702413104

Watson, A., El-Deredy, W., Iannetti, G. D., Lloyd, D., Tracey, I., Vogt, B. A., . . . Jones, A. K. P. (2009). Placebo conditioning and placebo analgesia modulate a common brain network during pain anticipation and perception. *Pain, 145*(1), 24-30. doi:10.1016/j.pain.2009.04.003

Wrobel, N., Wiech, K., Forkmann, K., Ritter, C., & Bingel, U. (2014). Haloperidol blocks dorsal striatum activity but not analgesia in a placebo paradigm. *Cortex, 57*, 60-73. doi:10.1016/j.cortex.2014.02.023

Yang, H., Yang, X., Liu, H., Long, H., Hu, H., Wang, Q., . . . Lai, W. (2021). Placebo modulation in orthodontic pain: a single-blind functional magnetic resonance study. *La radiologia medica, 126*(10), 1356-1365. doi:10.1007/s11547-021-01374-4

Zeidan, F., Emerson, N. M., Farris, S. R., Ray, J. N., Jung, Y., McHaffie, J. G., & Coghill, R. C. (2015). Mindfulness Meditation-Based Pain Relief Employs Different Neural Mechanisms Than Placebo and Sham Mindfulness Meditation-Induced Analgesia. *The Journal of Neuroscience, 35*(46), 15307-15325. doi:10.1523/jneurosci.2542-15.2015

Zeidan, F., Lobanov, O. V., Kraft, R. A., & Coghill, R. C. (2015). Brain mechanisms supporting violated expectations of pain. *Pain, 156*(9), 1772-1785. doi:10.1097/j.pain.0000000000000231

Zhang, W., Guo, J., Zhang, J., & Luo, J. (2013). Neural mechanism of placebo effects and cognitive reappraisal in emotion regulation. *Progress in Neuro-Psychopharmacology and Biological Psychiatry, 40*, 364-373. doi:10.1016/j.pnpbp.2012.10.020

Zhang, W., Qin, S., Guo, J., & Luo, J. (2011). A follow‐up fMRI study of a transferable placebo anxiolytic effect. *Psychophysiology, 48*(8), 1119-1128. doi:10.1111/j.1469-8986.2011.01178.x

Zhao, Y., Liu, R., Zhang, J., Luo, J., & Zhang, W. (2020). Placebo Effect on Modulating Empathic Pain: Reduced Activation in Posterior Insula. *Frontiers in Behavioral Neuroscience, 14*. doi:10.3389/fnbeh.2020.00008
